# Composite Anatomical Variations between the Sciatic Nerve and the Piriformis Muscle: A Nepalese Cadaveric Study

**DOI:** 10.1155/2020/7165818

**Published:** 2020-03-31

**Authors:** Ameet Kumar Jha, Prakash Baral

**Affiliations:** ^1^University of Central Nicaragua, Managua, Nicaragua; ^2^Department of Anatomy, Gandaki Medical College, Pokhara, Nepal

## Abstract

Piriformis syndrome is a rare syndrome which is one of the main causes of nondiscogenic sciatica causing severe low back pain due to entrapment of sciatic nerve either by the hypertrophy or by inflammation of the piriformis muscle. We have carried out dissection in 20 Nepalese cadavers. Out of 40 dissected gluteal regions, 37 exhibited typical appearance of sciatic nerve, piriformis muscle, and their relations resembling type-a, whereas 3 gluteal regions showed composite structural variations resembling type-b and type-c based on Beaton and Anson's classification. Knowledge pertaining to such variations will be helpful during a surgical intervention in the gluteal region and in turn reduces the risk of injuring these nerves which are more susceptible to damage. Our study reports such variations in Nepalese population which will be helpful during evaluation of the pain induction in various test positions and also useful for analysis of the range of the neurological deficiency in sciatic nerve neuropathies. The present study also explains the basis of the unsuccessful attempt of the sciatic nerve block during popliteal block anaesthesia.

## 1. Introduction

Sciatic nerve is the largest nerve carrying ventral rami of L4-S3 spinal nerves, which lies inferior to the piriformis muscle in the gluteal region [1]. It descends along the posterior aspect of the thigh and splits into the tibial and common peroneal components in the popliteal fossa [2, 3]. The provenance of the anatomical variation in pelvic region most likely originates from the existence of such structures as separate entities during the developmental period. Piriformis muscle originates from the pelvic surface of S2–S4 sacral segments, the superior margin of the greater sciatic notch, and the sacrotuberous ligament. Further, it exits through the greater sciatic foramen along with the sciatic nerve and inserts to the greater trochanter of the femur [1].

Sciatic nerve compression throughout its course can results into clinical conditions like sciatica and piriformis syndrome. Piriformis syndrome is a painful condition resembling sciatica, secondary to sciatic nerve entrapment, and is responsible for 6% of low back pain cases [4, 5]. It is one of the nondiscogenic causes of sciatica resulting due to the trauma, inflammation, and degenerative changes to the piriformis muscle. However, rare structural variations can be one of the main causes for this syndrome [6, 7]. It is essential to understand the anatomical variation of the sciatic nerve and piriformis muscle while performing the total hip arthroplasty, sciatic nerve block, and gluteal or pelvic surgery to avoid the iatrogenic injuries which may arise during these procedures [8]. Sciatic neuropathy results due to the compression or damage to the sciatic nerve, leading to the neurological deficits [9]. The structural variation of sciatic nerve, piriformis muscle, and their relations were first classified by Beaton and Anson into six different categories in 1937. Various researchers adopted this classification to categorize their findings. The two common groups of variations are the undivided sciatic nerve passing below unsplit muscle and variation where the sciatic nerve exits into the gluteal region through the greater sciatic foramen as two presplit branches, tibial and common peroneal nerve [8].

Beaton and Anson classified the anatomical relationship between the sciatic nerve and the piriformis muscle as follows:

  Type-a: undivided nerve below undivided muscle
  Type-b: divisions of nerve between and below undivided muscle
  Type-c: divisions above and below undivided muscle
  Type-d: undivided nerve between heads
  Type-e: divisions between and above heads
  Type-f: undivided nerve above undivided muscle [10, 11]

The aim of our study was to provide a compendious and evidence-based evaluation of composite anatomical variations of the sciatic nerve, piriformis muscle, and their relationship during routine dissection of Nepalese cadavers.

## 2. Methods

The current study was conducted on 40 gluteal regions of 20 formalin fixed human cadavers (18 males and 2 females) during routine dissection in the Anatomy Lab of Gandaki Medical College, Pokhara, Nepal.

The gluteal regions of these cadavers were carefully dissected followed by the retraction of the gluteus maximus muscle to expose the piriformis muscle and sciatic nerve. The sciatic nerve emerges inferior to the piriformis muscle and descends in the inferolateral direction. The variations in division of the sciatic nerve, abnormal origin of piriformis muscle, and their relations in all the specimens were carefully observed, noted, and photographed. Those structural variations were identified and classified based on the Beaton and Anson classification system. The pathological cadavers were excluded. No clinical data on them were available, and therefore, it was not known whether any of them had presented piriformis syndrome when alive. Some of these cadavers were unclaimed bodies, and therefore, the age groups were unknown; however, many of them were between the ages of 45 and 65 years.

We declare that appropriate ethical principles were followed during the procedures according to the regulations outlined in the Helsinki Declaration of the World Medical Association.

## 3. Case Series

In the current study, 37 (92.5%) out of 40 dissected gluteal regions of the human cadavers showed typical anatomical appearance of sciatic nerve, piriformis muscle, and their relations which resembled type-a based on Beaton and Anson's classification. However, three (7.5%) gluteal regions out of 40 showed composite structural variation of sciatic nerve, piriformis muscle, and their relations.

### 3.1. Case I

**Figure 1:**
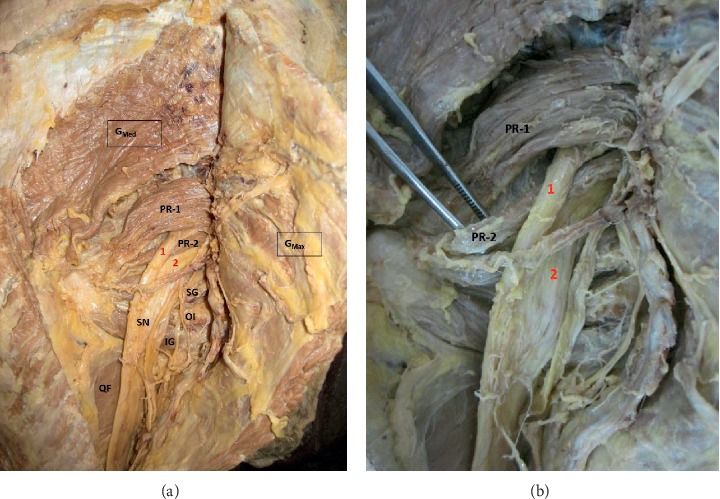
(a) Higher division of sciatic nerve and split piriformis muscle with two heads. 1: common peroneal superior head of piriformis; 2: tibial component; PR-1: inferior component; PR-2: head of piriformis; G_Max_: gluteus maximus; G_Med_: gluteus medius; SN: sciatic nerve; SG: superior gemellus. (b) Higher division of the sciatic nerve and split piriformis muscle with two heads. Forceps tip showing the inferior head of piriformis component. 1: common peroneal component; 2: tibial component; PR-1: inferior component; PR-2: head of piriformis.

A 54-year-old male cadaver during routine dissection showed composite piriformis muscle with peculiar arrangements of the two heads varying from its usual appearance and early splitting of the left sciatic nerve with tibial and common peroneal components (Figures 1(a) and 1(b)). The common peroneal component passes between the two heads of the piriformis muscle, whereas the tibial component had its usual course.

### 3.2. Case II

**Figure 2:**
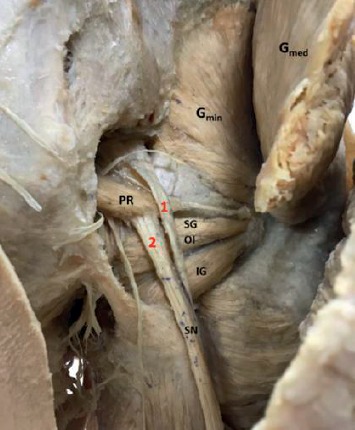
Higher division of sciatic nerve and a complete piriformis muscle. 1: common peroneal component; 2: tibial component; PR: piriformis muscle; G_med_ gluteus medius; G_min_: gluteus minimus; SN: sciatic nerve; SG: superior gemellus.

A 47-year-old male cadaver during similar routine dissection showed early splitting of the right sciatic nerve where the common peroneal component descends posterolateral to the piriformis muscle, whereas the tibial component had its usual course (Figure 2). The result corresponded with Beaton and Anson type-c (2.5%).

### 3.3. Case III

A similar unilateral variation in the formation of sciatic nerve and its unusual relation with the piriformis muscle was encountered in the left gluteal region of a 51-year-old male cadaver. Additionally, an accessory belly of the piriformis muscle at its insertion was observed in the right gluteal region beneath the gluteus maximus of the same cadaver. The relationship of the sciatic nerve and piriformis muscle in this case was classified under type-c (2.5%). There were no any gross anomalic appearance or any surgical intervention in these cadavers near the dissected area.

## 4. Discussion

Piriformis syndrome is considered to be an atypical, contentious neuromuscular disorder resulted from a compression of the sciatic nerve at the level of the piriformis muscle. The diagnosis has been a major challenge due to difficulties in finding the exact cause of the pain and a paucity of confirmed clinical and definitive diagnostic criteria like radioimaging or electrodiagnostic testing [6, 13].

The anomalic variation of sciatic nerve, piriformis muscle, and their variable relationship can lead to entrapment and compression of the nerve, resulting into piriformis syndrome [14]. Such variations have to be emphasized as it plays a crucial role in the basis of sciatica and the pain etiology [12]. The branching patterns of the two main divisions of the sciatic nerve, the tibial, and the common fibular nerve are responsible for these variations [7].

Piriformis syndrome narrates the presence of pain in the buttock and posterior hip region caused from nondiscogenic and extrapelvic entrapment of the sciatic nerve [15]. The current study aims to explore the Nepalese cadavers to provide awareness and strengthen the findings of the sciatic nerve variations and its relation to the piriformis as a probable cause for the nondiscogenic sciatica as well as other pain etiologies.

In the present study, we have encountered 37 (92.5%) type-a, 1 (2.5%) type-b, and 2 (5%) type-c nerve-muscle relations out of 40 dissected gluteal regions in the Nepalese cadavers (Table 1). Adibatti et al. reported a similar finding with 92% of type-a, 2% of type-b, and 6% of type-c with dissection on 50 gluteal regions of the Indian population [14]. Desalegn et al. found 91.7% of type-a, 2.8% of type-b, and 5.5% of type-c while dissecting 36 gluteal regions of the northern Ethiopian population [16]. A similar study on 30 Polish cadavers revealed 76.7% of type-a, 20% of type-b, and 3.3% of type-c [17]. Berihu et al. with their research on 56 Ethiopian cadavers marked 89.3% of type-a, 8.9% of type-b and 1.8% of type-c relations [2].

Güvençer et al., Patel et al., Brooks et al., and Ogeng'o et al., with their research on Turkish, Indian, Brazilian, and Kenyan cadavers reported 76%, 91.9%, 90%, and 89.7% type-a relations and 24%, 8.1%, 10%, and 10.3% of type b-f relations, respectively [18–21].

**Figure 3:**
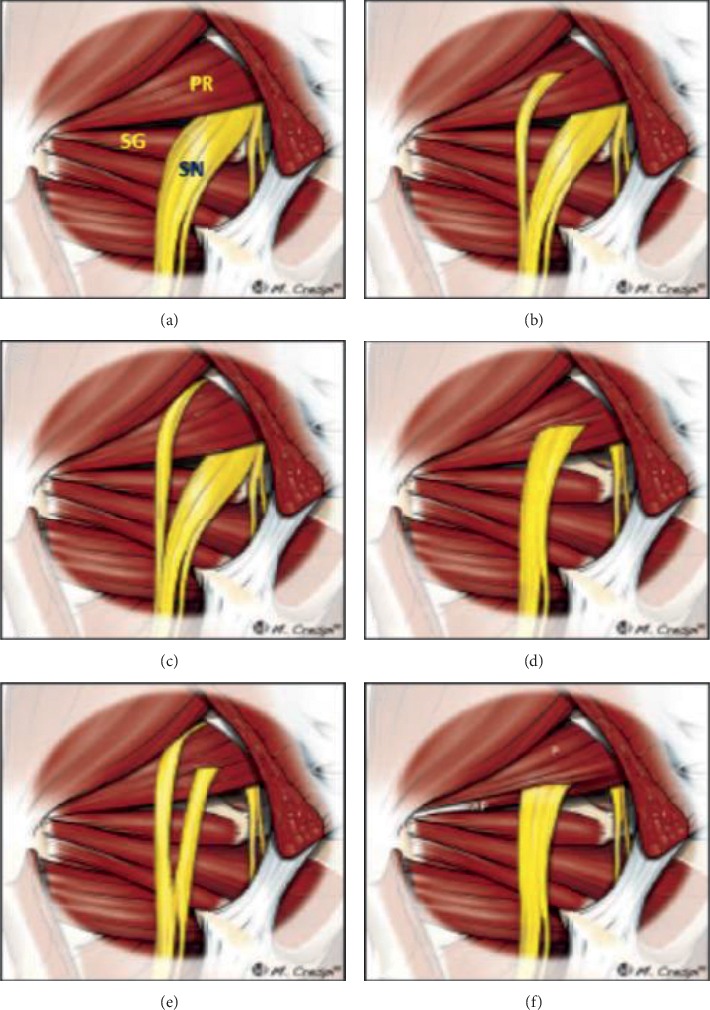
Anatomic variations of the relationship between the piriformis muscle and sciatic nerve. Diagrams illustrate the six variants, originally described by Beaton and Anson. (a) An undivided nerve comes out below the piriformis muscle (normal course). (b) A divided sciatic nerve passing through and below the piriformis muscle. (c) A divided nerve passing above and below an undivided muscle. (d) An undivided sciatic nerve passing through the piriformis muscle. (e) A divided nerve passing through and above the muscle heads. (f) Diagram showing an unreported additional variation of d-type consisting of an accessory piriformis (AP) muscle head with its own separate tendon. SN: sciatic nerve; PR: piriformis muscle; SG: superior gemellus [12].

An extensive research on the Czech Republican cadavers revealed 79.1% type-a, 14.3% type-b, 4.4% type-c, and 2.2% type-d relationship between the sciatic nerve and piriformis muscle [22]. Almost a similar type of finding was noted by Sinha et al. who researched on 100 Indian cadavers and showed 85% type-a, 9% type-b, 3% type-c, and 3% type-d relations [23]. Pokorny et al., Brooks et al., Natsis et al., and Sinha et al., also found 2.2%, 10%, 0.35%, and 3% type-d relations, respectively [7, 20, 22, 23]. Natsis et al., with their research on 290 Greek cadavers revealed 0.35% extremely rare type-f relation [7]. However, the current study did not find any type-d and type-f relations probably due to limitation of small sample size (Figure 3).

Studies on 24 Malaysian and 102 American cadavers have reported 83.4% and 88.2% type-a relations and 16.6% and 11.8% type b-f relations, respectively; however, type-d, type-e, and type-f relations were absent, which was also noticed in current study [24, 25]. A research on 120 American cadavers detected 97.5% type-a and 2.5% type-b relations, which was almost similar to our study [12].

This relationship of sciatic nerve and piriformis muscle was classified as type-b (2.5%). However, in this case, we also observed an altered anatomical variation where a higher division of the sciatic nerve occurs and the common peroneal component passes between the two heads of the piriformis muscle.

A substantial research on 400 fetal cadavers in Turkey revealed 98% type-a 1.2% type b, and 0.8% type c relations [29]. The current study showed similar results; however, the fetal cadavers were not included due to unavailability.

The probability of anatomical variation in cadavers makes it essential among clinicians and surgeons to be aware of the potential complications during medical or surgical interventions [30].

There are several potential etiologies of posterior hip and buttock pain. Jonathan et al. focused on the structural variation that plays a significant role in causing piriformis syndrome; however, the variations unassociated to piriformis syndrome were ruled out due to the difficulty in their identification [12]. In some rare circumstances, anatomic variations may be the source of refractory sciatic pain. Although the variations were recognized in the early 1900s, these findings are uncommon and not readily seen on diagnostic imaging studies. Diagnosis for this syndrome has been historically problematic due to difficulties finding objective evidence as the source of pain. It is usually a diagnosis of exclusion which is made by clinical findings [31]. Neuroimaging techniques are emerging and gaining its popularity, and therefore, researchers and neurophysicians will be able to benefit more precisely in recognizing, diagnosing, treating, and managing the pain associated with nerve entrapment due to composite piriformis-sciatic nerve anomalies. The present study provides awareness of additional sciatic nerve entrapments that are possible within the gluteal regions in the Nepalese population. As a future implication, it is important to emphasize on the study of the embryological basis of these structural variations and their origins.

## 5. Conclusion

It is important to be aware of anatomical variation in sciatic nerve during a surgical intervention in the gluteal region so as to reduce the risk of injuring these nerves which are more susceptible to be injured. A detailed anatomical study of such variations will be helpful for evaluating the pain in various test positions. The present study provides awareness of additional sciatic nerve entrapments that are possible within the gluteal regions in the Nepalese population. This study is useful for analysis of the range of the neurological deficiency in sciatic nerve neuropathies and explains the basis of the unsuccessful attempt of the sciatic nerve block during popliteal block anaesthesia.

## 6. Limitations

One of the limitations of this study is small sample size. Therefore, increased sample size has to be considered in future studies. Our research lacks the information on family and medical history of the donors which would have revealed more inference in pain etiology and management.

## Figures and Tables

**Table 1:** Summary of sex-wise distribution of dissected gluteal regions and their classification based on Beaton and Anson.

Sex	No. of gluteal regions dissected		Type-a		Type-b		Type-c	
	Right	Left	Right	Left	Right	Left	Right	Left
Male	18	18	17	16	0	1	1	1
Female	2	2	2	2	0	0	0	0

## References

[1] Moore K. L., Dalley A. F., Agur A. M. R. (2018). *Clinically Oriented Anatomy*.

[2] Berihu B. A., Debeb Y. G. (2015). Anatomical variation in bifurcation & trifurcations of sciatic nerve & its clinical implications: in selected university in Ethiopia. *BMC Research Notes*.

[3] U.S. Department of Health and Human Services. Health, United States, Centers for Disease Control and Prevention, 2006, https://www.cdc.gov/nchs/data/hus/hus06.pdf

[4] Mitra S. R., Roy S., Dutta A. S., Ghosh A., Roy R., Jha A. K. (2014). Piriformis syndrome: a review. *Journal of Evolution of Medical and Dental Sciences*.

[5] Yeoman W. (1928). The relation of arthritis of the sacro-iliac joint to sciatica, with an analysis of 100 cases. *The Lancet*.

[6] Ro T. H., Edmonds L. (2018). Diagnosis and management of piriformis syndrome: a rare anatomic variant analyzed by magnetic resonance imaging. *Journal of Clinical Imaging Science*.

[7] Natsis K., Totlis T., Konstantinidis G. A., Paraskevas G., Piagkou M., Koebke J. (2014). Anatomical variations between the sciatic nerve and the piriformis muscle: a contribution to surgical anatomy in piriformis syndrome. *Surgical and Radiologic Anatomy*.

[8] Tomaszewski K. A., Graves M. J., Henry B. M. (2016). Surgical anatomy of the sciatic nerve: a meta-analysis. *Journal of Orthopaedic Research*.

[9] Prakash B. A. K., Devi M. N., Sridevi N. S., Rao P. K., Singh G. (2010). Sciatic nerve division: a cadaver study in the Indian population and review of literature. *Singapore Medical Journal*.

[10] Beaton L. E., Anson B. J. (1937). The relation of the sciatic nerve and of its subdivisions to the piriformis muscle. *The Anatomical Record*.

[11] Beaton L. E. (1938). The sciatic nerve and piriform muscle: their interrelations possible cause of coccygodynia. *J Bone Joint Surg Am*.

[12] Van Erdewyk, Jonathan I. (2017). Anatomical variations of the sciatic nerve divisions in relation to the piriformis muscle and clinical implications. http://digitalcommons.unmc.edu/etd/194.

[13] Miller T. A., White K. P., Ross D. C. (2012). The diagnosis and management of piriformis syndrome: myths and facts. *Canadian Journal of Neurological Sciences/Journal Canadien des Sciences Neurologiques*.

[14] Adibatti M. (2014). Study on variant anatomy of sciatic nerve. *Journal of Clinical and Diagnostic Research : JCDR*.

[15] Kulcu D. G., Naderi S. (2008). Differential diagnosis of intraspinal and extraspinal non-discogenic sciatica. *Journal of Clinical Neuroscience*.

[16] Desalegn M., Tesfay A. (2014). Variations of sciatic nerve its exit in relation to piriformis muscle in the northern Ethiopia. *International Journal of Pharmaceutical Sciences and Research*.

[17] Haladaj R., Pingot M., Polguj M., Wysiadecki G., Topol M. (2015). Anthropometric study of the piriformis muscle and sciatic nerve: a morphological analysis in a polish population. *Medical Science Monitor: International Medical Journal of Experimental and Clinical Research*.

[18] Güvençer M., Iyem C., Akyer P., Tetik S., Naderi S. (2009). Variations in the high division of the sciatic nerve and relationship between the sciatic nerve and the piriformis. *Turkish Neurosurgery*.

[19] Patel S., Shah M., Vora R., Zalawadia A., Rathod S. P. (2011). A variation in the high division of the sciatic nerve and its relation with piriformis muscle. *National Journal of Medical Research*.

[20] Brooks J. B. B., Silva C. A. C., Soares S. A., Kai M. R., Cabral R. H., Fragoso Y. D. (2011). Variações anatômicas do nervo ciático em um grupo de cadáveres brasileiros. *Revista Dor*.

[21] Ogeng’o J. A., El-Busaidy H., Mwika P. M., Khanbhai M. M., Munguti J. (2016). Variant anatomy of sciatic nerve in a black Kenyan population. *Folia Morphol*.

[22] Pokorný D., Jahoda D., Veigl D., Pinskerová V., Sosna A. (2006). Topographic variations of the relationship of the sciatic nerve and the piriformis muscle and its relevance to palsy after total hip arthroplasty. *Surgical and Radiologic Anatomy*.

[23] Sinha M. B., Aggarwal A., Sahni D., Harjeet K., Gupta R., Sinha H. P. (2014). Morphological variations of sciatic nerve and piriformis muscle in gluteal region during fetal period. *European Journal of Anatomy*.

[24] Khan A. A., Asari M. A., Pasha M. A. (2016). The sciatic nerve in human cadavers-high division or low formation?. *Folia Morphologica*.

[25] Lewis S., Jurak J., Lee C., Lewis R., Gest T. (2016). Anatomical variations of the sciatic nerve, in relation to the piriformis muscle. *Translational Research in Anatomy*.

[26] Singh A. K., Sharma R. C. (2011). Relationship between the sciatic nerve and piriformis muscle. *Neuroscience Research Letters*.

[27] Shewale A. D., Karambelkar R. R., Umarji B. N. (2013). Study of variations in the divisions, course and termination of the sciatic nerve. *Journal of Krishna Institute of Medical Sciences University*.

[28] Muthu Kumar T., Srimathi, Rani A., Latha S. (2011). A cadaveric study of sciatic nerve and it’s level of bifurcation. *Journal of Clinical and Diagnostic Research*.

[29] Sulak O., Sakalli B., Ozguner G., Kastamoni Y. (2014). Anatomical relation between sciatic nerve and piriformis muscle and its bifurcation level during fetal period in human. *Surgical and Radiologic Anatomy*.

[30] Smoll N. R. (2010). Variations of the piriformis and sciatic nerve with clinical consequence: a review. *Clinical Anatomy*.

[31] Cassidy L., Walters A., Bubb K. (2012). Piriformis syndrome: implications of anatomical variations, diagnostic techniques, and treatment options. *Surgical and Radiologic Anatomy*.

